# Mother’s perceptions of child mental health problems and services: A cross sectional study from Lahore

**DOI:** 10.12669/pjms.323.9775

**Published:** 2016

**Authors:** Nazish Imran, Sania Ashraf, Rabia Shoukat, Muhammad Ijaz Pervez

**Affiliations:** 1 Dr. Nazish Imran, MBBS; MRCPsych (London). Associate Professor, Child & Family Psychiatry Department, King Edward Medical University/Mayo Hospital, Lahore, Pakistan; 2 Dr. Sania Ashraf, Resident Medical Officer. Child & Family Psychiatry Department, King Edward Medical University/Mayo Hospital, Lahore, Pakistan; 3 Miss Rabia Shoukat, Intern Psychologist, Department of Psychiatry & Behavioural Sciences, King Edward Medical University/Mayo Hospital, Lahore, Pakistan; 4 Dr. Muhammad Ijaz Pervez, MBBS. House Officer, Academic Department of Psychiatry & Behavioural Sciences, King Edward Medical University/Mayo Hospital, Lahore, Pakistan

**Keywords:** Mothers, Perceptions, Mental health, Emotional, behavioural problems, Pakistan

## Abstract

**Objective:**

To assess the perceptions of mothers regarding child mental health problems, its causes, preferred treatment options, and to determine whom they would consult, if their child had a psychiatric illness.

**Methods:**

Following informed consent, a questionnaire covering perceptions regarding various aspects of child mental illness was used for data collection from mothers. They were asked to identify the symptoms and behaviours they considered psychopathological in children, which treatments they would prefer, where they would turn for help with a mentally ill child, and their understanding of the causes of child psychiatric disorders in addition to ways to increase awareness of child psychiatric issues in the society.

**Results:**

Ninety one mothers participated in the study. They equally perceived emotional, behavioural and cognitive symptoms as suggestive of mental ill health in childhood. Mothers perceived multiple causes of child mental health problems, including family problems, economic difficulties, social adversity and possession by evil spirits. A substantial proportion preferred medication, recitation of Holy Quran and psychotherapy as the preferred treatment options. Overall, mothers preferred consulting health professionals than religious scholars and faith healers. They were keen for steps to increase mental health awareness within their society.

**Conclusion:**

Despite different cultural perspective, mothers exhibit good understanding of symptoms of child mental health issues and appear open to various services and treatment options. Understanding parental perceptions and expectations from child psychiatric services are crucial in increasing families’ engagement in treatment.

## INTRODUCTION

Despite reports of significantly higher prevalence of emotional and behavioural problems in children in Pakistan, only a limited number of these children receive any psychiatric services.^1^,^2^ One needs to review the possible barriers to utilization of services as children rarely make their own treatment decisions, Alongside stigma, economic adversity & limited child & adolescent mental health services, socio-cultural factors and parental perceptions are being increasingly recognized as important in recognition and presentation of child mental health disorders and use of services.^3^ Studies suggests differences among parents of various cultural groups in their expectations of children behaviours, beliefs about causes and appropriate treatments etc. with over reporting of behavioural problems while emotional problems remains underreported.^4^,^5^

In view of high prevalence of child psychiatric disorders in Pakistan, there is an urgent need to provide culturally acceptable services to children with mental illness however we do not know which behavioural and emotional problems are considered as child psychiatric disorders by the parents, or how they conceptualize the causes of mental illness neither are we aware who they would go to, if they thought that their child has a psychiatric disorder. To address this knowledge gap, a cross sectional survey was conducted to assess mother’s perceptions of child mental health problems and services in a tertiary care hospital in Lahore.

## METHODS

Participants were mothers of children presenting to Mayo hospital Paediatrics outpatient department for minor physical ailments. They were informed regarding the study’s purpose, confidentiality issues etc. Following written informed consent, data was collected using a questionnaire, which had two sections. First part collected demographic information like age, education etc. Second section included a checklist derived from literature in which mothers were asked to identify the symptoms or behaviours, which they consider as psychopathological, their understanding of the causes of child psychiatric disorders, their views about the interventions for these problems and where they would turn for help with a mentally ill child.^3^ They were also asked suggestions about ways to increase awareness of child psychiatric problems in the society. The questionnaire was administered in an interview format due to literacy issues. Data was analyzed using SPSS 17.

## RESULTS

Ninety one mothers among the 105 approached, agreed to participate. Only reasons given for refusing to participate were hurry to return home or child being upset/crying due to being in hospital environment. Mean age was 32.8±6.36. A significant majority of mothers 82(91%) were house wives. Seventy seven (84.6%) had education below matriculation and 75(82.4%) stated their monthly household income as less than 20,000 rupees.

How frequently the various emotional, cognitive and behavioural manifestations were perceived by the mothers as mental health problems in a child are shown in Table-I. When asked about their views on the causes of child mental health problems, most mothers reported multiple causes including family problems, socio economic reasons, genetic causes, being possessed etc. (Table-II)

**Table-I:** Frequency of mothers perceiving various emotional, cognitive & behavioural manifestations as mental health problems (N= 91).

*Mental Health problems Manifestations*	*N (%)*
***Emotional & Cognitive Manifestations***	
Suicidal Thoughts	60(65.9)
Phobias	58(63.7)
Hallucinations	56(61.5)
Depression	43(47.3)
School refusal	39(42.9)
Inattention	37(40.7)
False beliefs	32(35.2)
Somatic complaints	26(28.6)
Daytime wetting of clothes	16(17.6)
***Behavioural Manifestations***	
Outbursts of anger	56(61.5)
Destructive Behaviour	52(57.1)
Disobedience	47(51.6)
Verbal abuse	46(50.5)
Fighting	44(48.4)
Physical abuse of others	43(47.3)
Lying	41(45.1)
Fire setting	37(40.7)
Sleep problems	31(34.1)
Escape from home	28(30.8)
Drug use.	23(25.3)
Truancy from school	21(23.1)

*Values differ depending on the number of respondents who answered particular questions, (i.e. due to some missing data).

**Table-II:** Mother’s views regarding likely causes and preferred treatment for mental disorders.

	*N (%)*
***Causes of Mental Health Problems***	
Family problems	60(65.9)
Social problems	55(60.4)
Economic problems	54(59.3)
Genetic diseases	39(42.9)
Being possessed	33(36.3)
Organic brain lesions	27(29.7)
Others	8(8.8)
***Preferred type of treatment***	
Medication	70(76.9)
Recitation of Holy Quran	60(65.9)
Psychotherapy	52(57.1)
Taveez	14(15.4)

*Values do not add up to 100% as respondents were allowed to choose more than one option.

Seventy one mothers would take their child to psychiatrist or psychologist if concerned about their child having any mental health problems; 40(44%) would consult a paediatrician, 25(27.5%) a religious scholar, 22(24.2%) a general practitioner 16(17.6%) a spiritual healer while 11(12.1%) would take their child to homeopathic doctor for their child mental health issues. Medication were the most preferred treatment endorsed by majority of mothers as treatment for child mental health problems. (Table-II)

In response to question about the sources which might be helpful to mothers to understand child mental health problems better, 46(50.5%) respondents suggested TV programmes directed at children and adolescents mental health problems, regular leaflets containing information and advice about child mental health problems (42; 46.2%), Public meetings between parents and professionals (41, 45.1%) & series of lectures for teachers and parents (40, 44%).

## DISCUSSION

Our study explored the perceptions of mothers related to child mental illnesses and services in Lahore. Mothers perceived equally, the emotional, behavioural and cognitive symptoms as possible representations of child psychiatric disorders. This is in line with results reported from the Arab World.^3^ However this equal emphasis on emotional and behavioural symptoms is in contrast with differential reporting of these symptoms in earlier studies of parents perceptions.^4^,^5^ Surprisingly only a small proportion of our respondents endorsed somatic complaints as a possible symptoms of child psychiatric illness despite evidence showing a significant association between unexplained somatic symptoms and anxiety and depression in Pakistani children.^6^

Several possible aetiological factors for child mental illness were considered by mothers with family, social and economic factors given highest importance. Adolescents own emotional state, stress, family problems and bad luck were endorsed as some of the causal factors in their mental illness by Pakistani adolescents.^7^ A substantial proportion also mentioned possession by evil spirits as a possible cause. Chemical imbalance, traumatic experiences, spiritual reasons were endorsed as most likely causes for child mental illness in a study of 283 Vietnamese parents in Australia. Only a small minority indicated “Karma” to be the possible cause.^8^ Similarly Possession by demons, punishment inflicted by God, Fate, and black magic are some of the etiological factors for Mental illness reported mostly in Asian & Arab countries.^9^

Mothers in our study would prefer to contact a professional person (Psychiatrist, psychologist, Paediatrician) rather than a religious scholar or spiritual healer. This finding most likely is a reflection of gradually increasing awareness of child mental health issues in Pakistan, although stigma, myths in society still means significant delay in accessing the available services. Similar trend of preference of consulting health professionals and modern modalities of treatments rather than traditional ones is seen in other studies as well.^3^,^8^ “Medications” and “talking treatment” were preferred interventions by most mothers for child psychiatric illness but a considerable proportion (66%) would also consider “recitation from Holy Quran”. This may be due to Religion/Islam being, crucial in all aspects of life in Pakistani society. Parental perceptions of perceived benefits of treatment has been observed to be strongly related to compliance and engagement with services.^10^,^11^ Another observation was limited involvement of fathers in help seeking for their children’s health issues. It may be due to cultural and societal factors making childcare primarily a mothers responsibility or may be that services in public sector hospitals are offered mainly in morning and fathers due to being primary breadwinner cannot lose a day of earning in order to bring children to hospital. This issue may be explored in detail in further studies.

The study has many limitations like small sample size and representation from only one centre. Very few fathers were observed accompanying the children, thus we focused on data collection from mothers only. Also semi structured interviews are considered more helpful to explore perceptions and belief system. Despite study limitations we believe that our study shed light on an important and poorly understood area i.e. mothers understanding of likely causes, symptoms and most appropriate interventions for child psychiatric illness. Further qualitative studies can be planned in future to get a broader understanding of parental perceptions in order to help plan culturally appropriate services for vulnerable children.
